# A Wireless Magnetic Resonance Energy Transfer System for Micro Implantable Medical Sensors

**DOI:** 10.3390/s120810292

**Published:** 2012-07-30

**Authors:** Xiuhan Li, Hanru Zhang, Fei Peng, Yang Li, Tianyang Yang, Bo Wang, Dongming Fang

**Affiliations:** 1 School of Electronics and Information Engineering, Beijing Jiaotong University, Beijing 100044, China; E-Mails: hanruzhang@126.com (H.Z.); 11120030@bjtu.edu.cn (F.P.); airflyfaith@163.com (Y.L.); 10120044@bjtu.edu.cn (T.Y.); wangbotju@126.com (B.W.); 2 State Key Laboratory of Transducer Technology, Institute of Electronics, Chinese Academy of Sciences, Beijing 100190, China; E-Mail: fangdm@pku.edu.cn

**Keywords:** wireless energy transfer system, transfer efficiency, Class-E amplifier, CMOS rectifier, LDO, micro-implantable medical sensors

## Abstract

Based on the magnetic resonance coupling principle, in this paper a wireless energy transfer system is designed and implemented for the power supply of micro-implantable medical sensors. The entire system is composed of the *in vitro* part, including the energy transmitting circuit and resonant transmitter coils, and *in vivo* part, including the micro resonant receiver coils and signal shaping chip which includes the rectifier module and LDO voltage regulator module. Transmitter and receiver coils are wound by Litz wire, and the diameter of the receiver coils is just 1.9 cm. The energy transfer efficiency of the four-coil system is greatly improved compared to the conventional two-coil system. When the distance between the transmitter coils and the receiver coils is 1.5 cm, the transfer efficiency is 85% at the frequency of 742 kHz. The power transfer efficiency can be optimized by adding magnetic enhanced resonators. The receiving voltage signal is converted to a stable output voltage of 3.3 V and a current of 10 mA at the distance of 2 cm. In addition, the output current varies with changes in the distance. The whole implanted part is packaged with PDMS of excellent biocompatibility and the volume of it is about 1 cm^3^.

## 1. Introduction

Micro-implantable medical devices (IMDs) are becoming more and more popular in health and medical applications due to the ability to locally stimulate internal organs and communicate the internal vital signs to the outer world. The internal battery is not an ideal candidate for the power supply due to its limited life time, large volume, and possibility of leakage [1]. Energy harvesters [2] which can capture mechanical, thermal, chemical, gravitational or electromagnetic energy had at one time been considered as a suitable power supply method for IMDs, but they can't work well without a proper environmental energy source [3].

Recently, wireless power transfer schemes have often used in IMDs to not only to avoid transcutaneous wiring, but also to either recharge or replace the device battery. Wireless energy transfer can be divided into the near field and the far field transmission. The far field radiative transfer by microwaves or laser is limited by absorption and scattering in the atmosphere and requires a direct line of sight between the source and the device(s) [4]. At high intensities, it also presents a challenging electromagnetic interference problem. The near-field transmission includes inductive coupling and magnetic resonance coupling. Inductive coupling energy transfer through the mutual inductance between two coils was first used to power IMDs [5]. In these systems, the efficiency is affected by many factors such as the size, structure physical spacing, relative location, and the properties of the environment surrounding the coils. The coupling between the two coils decreases sharply as the distance (d) between the coils increases and causes the overall power transfer efficiency to decrease monotonically [6]. The geometric mean r_m_ (*r*_m_ = √*r*_1_ × *r*_2_) of the transmitter and receiver coil radii (r_1_, r_2_) is commonly used as the performance metric for comparing different designs. The inductive coupling efficiency is lower than 40% and decreased with 1/d^3^ when d > r_m_ [7].

In 2007, Kurs reported [8] a new wireless power transfer system based on strongly magnetic resonant coupling between four coils according to coupled mode theory (CMT) [9], and a transmission efficiency of 40%∼50% was obtained in the middle range of 2.74 m (a few times of the coil size). Distinct from the inductive coupling, an intuitive but powerful concept is employed. For instance, two objects with the same intrinsic resonant frequency exchange energy more efficiently than two non-resonant objects. Another attractive feature is that magnetic fields interact with living organisms weakly, making it a relatively safer method for energy transfer than the RF methods which involve interactions of both electric and magnetic fields with biological tissues. Although The MIT system has obvious advantages in the transmission efficiency and distance, the coil size (for 60 cm in diameter) is not suitable for IMDs. Magnetic resonance coupling with centimeter-scale size of coils has been designed in order to promote the application of magnetic resonance energy coupling in IMDs. Sun *et al.* [10,11] designed a wireless energy transfer system with transmitter coils (165 mm in diameter) and receiver coils (41 mm in diameter) based on CMT. The efficiency is 4.67% at the distance of 70 cm and 26.8% at the distance of 33 cm. Since CMT is founded on the magnetic resonant coupling between two ideal models with an identical and big size, it is not suitable for coupling coils with great size disparity. Chiao [12] uses resonant circuit theory to analyze the magnetic resonating system. The coupling coils are wounded by Litz wire, the diameters of transmitter coils and receiver coils are 64 mm and 22 mm, respectively. The energy transfer efficiency of 82% is achieved at the distance of 20 mm (r_m_/d = 1.07). It is proved that the efficiency of resonant coupling is much higher than that of inductive coupling.

**Figure 1.**
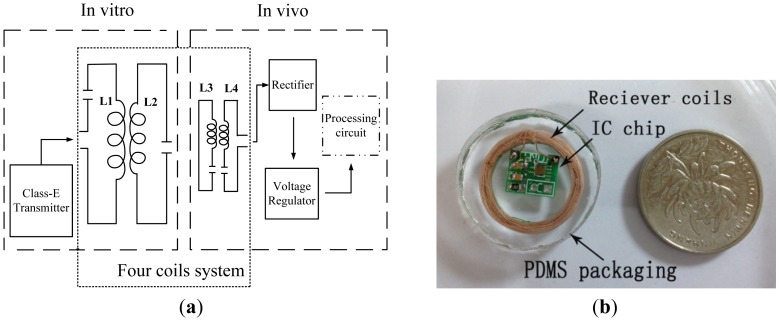
(**a**) Schematic of the wireless resonant energy transfer system; (**b**) Packaged implanted receiver coils and IC chip by PDMS.

Based on the four-coil resonant circuit theory, a wireless energy transfer system (Figure 1(a)) is designed and implemented in this paper. The entire system is composed of the *in vitro* part, including the energy transmitting circuit (Class-E amplifier) and resonant transmitter coils, which consist of drive coil (coil1) and primary coil (coil2), and the *in vivo* part including the micro resonant receiver coils which consist of the secondary coil (coil3) and load coil (coil4) and signal shaping chip which includes the rectifier module and LDO voltage regulator module. First the electrical and geometric parameters of the coils are theoretically optimized. Then the energy transfer efficiency is modeled and optimized. The peripheral circuit is composed of Class-E amplifier, low power CMOS rectifier circuit, and capacitor-less low dropout linear voltage regulator (LDO). Finally, an experimental setup is designed to characterize the energy transfer efficiency. Experimental results show that the energy transfer efficiency of resonant four coils is much higher than that of two coils. When the distance between the transmitter coils and the receiver coils is 1.5 cm and the carrier frequency is 742 kHz, the transfer efficiency is 85%. What's more, the power transfer efficiency can be optimized by adding magnetic enhanced resonators. The whole implanted micro system is packaged with polydimethylsiloxane (PDMS) after depositing a layer of Parylene to obtain better seal and biocompatibility as shown in Figure 1(b). The measurement results show that the receiving voltage signal is converted to a stable output voltage of 3.3 V and a current of 10 mA at the distance of 2 cm. A power of more than 100 mW can be achieved when the distance is decreased, which can meet the power consumption requirements of most reported biomedical systems such as artificial retinas (power consumption of 42 mW) [13], intraocular pressure sensors (power consumption of μWs or mWs) [14], neural recording systems (power consumption of 5.3 mW) [15], and human body sensor networks [16].

This paper is organized as follows: the models of resonant coils and energy transfer efficiency are calculated and optimized in Section 2. Section 3 describes the design of peripheral circuit including the transmitter module and receiver module of the wireless power transfer system. The experimental setup and measurement results about the energy transfer efficiency and the whole system are presented and discussed in Section 4.

## 2. Transcutaneous Magnetic Resonance Power Link Design

Energy transfer efficiency is the key issue for the four-coil resonant power link. It depends on the self-inductance, quality factor (Q factor) and the carrier frequency. The coils are wound with Litz wire in order to reduce the ac resistance and improve the Q factor. First, a theoretical model for designing transmitter and receiver coils based on multistrand Litz wire is applied.

### 2.1. Optimization of Q Factor for the Resonant Coils

In order to reduce and utilize the volume effectively, a multilayer solenoidal coil model is chosen. In addition, transmitter coils are composed of coil2 wrapped over coil1, and receiver coils are composed of coil3 wrapped over coil4. The inductance is derived by the summation of each turns' inductance and the mutual inductance between each turn.

For a solenoidal coil with *N_a_* layers, *N_t_* turns per layer and different radii *a_i_* (*i* = 1, 2, …, *N_a_*), wire radius *R*, the total self-inductance can be modeled as [6]:

(1) $$L_{a}=N_{t}\sum_{i=1}^{Na}\mu_{0}\ln\left(\frac{8a_{i}}{R}-2\right)+\sum_{i=1}^{Na}\sum_{j=1}^{Na}\sum_{k=1}^{Nt}\sum_{l=1}^{Nt}M(a_{ik},a_{jl},\rho =0,d=d_{l}\left|k-l\right|)\times (1-\delta_{ij})(1-\delta_{kl})$$

where *δ_ij_* (or *δ_kl_*) = 1 for i = j (or *k* = *l*) and *δ_ij_* (or *δ_kl_*) = 0 otherwise. *d_l_* is the minimum distance between two consecutive turns, *μ_0_* is the permeability of vacuum. The first half of Equation (1) represents the summation of each turns' inductance, and the second half of Equation (1) represents the summation of mutual inductance between each turn, which can be calculated as [17,18]:

(2) $$M(a,b,\rho =0,d)=\mu_{0}\sqrt{ab}\left[\left(\frac{2}{k}-k)K(k)-\frac{2}{k}E(k\right)\right]$$

(3) $$k={\left(\frac{4ab}{{\left(a+b\right)}^{2}+d^{2}}\right)}^{1/2}$$

where *K*(*k*) and *E*(*k*) are the complete elliptic integrals of the first and second kind, *a* and *b* are the radii of two single-turn coils.

To achieve a high Q factor, consideration of different parameters such as the self-inductance of coil and operating frequency (*f*) is required. Q factor can be captured as [6]:

(4) $$Q=\frac{2\pi fL_{\text{self}}(1-\frac{f^{2}}{{f_{\text{self}}}^{2}})}{R_{dc}(1+\frac{f^{2}}{{f_{h}}^{2}})}$$

The self-resonance frequency *f_self_* can be derived by the self-inductance (*L_a_*) and parasitic capacitance (*C_self_*) of the coil:

(5) $$f_{\text{self}}=\frac{1}{2\pi \sqrt{L_{a}C_{\text{self}}}}$$

For a multilayer solenoidal coil with *N_a_* layers and *N_t_* turns per layer, parasitic capacitance (*C_self_*) can be calculated as [19]:

(6) $$C_{\text{self}}=\frac{1}{N^{2}}\left[C_{b}(N_{t}-1)N_{a}+C_{m}\sum_{i=1}^{N_{t}}{\left(2i-1\right)}^{2}(N_{a}-1)\right]$$

where *N* is the total turns, *C_b_* is the parasitic capacitance between two nearby turns in the same layer, and *C_m_* is the parasitic capacitance between different layers.

The ac resistance of coils made up of multistrand Litz wires, including skin and proximity effect, can be approximated as [20]:

(7) $$R_{ac}=R_{dc}(1+\frac{f^{2}}{f_{h}^{2}})$$

where *f_h_* is the frequency at which power dissipation is twice the dc power dissipation and *R_dc_* is the dc resistance of the coil, which are given as Equation (8) [20] and Equation (9) [12]:

(8) $$f_{h}=\frac{2\sqrt{2}}{\pi r_{s}^{2}\mu_{0}\sigma \sqrt{NN_{s}\eta_{a}\;\beta }}$$

(9) $$R_{dc}=\sum_{i=1}^{Na}\pi N_{t}D_{i}\frac{\rho R_{s}{\left(1.015\right)}^{N_{B}}{\left(1.025\right)}^{N_{C}}}{AN_{s}}$$

where *r_s_, N_s_, β* are the radius of each single strand, number of strands per bunch, and the area efficiency of the bunch, respectively. η_a_ is the area efficiency of coil with width b and thickness t. *A, R_S_, N_B_, N_C_*, and *N_S_* are the cross-section area, maximum dc resistance of each individual strand, number of bunching operations, number of cabling operations, and number of individual strands, respectively.

At high frequency, skin and proximity effect increase the ac resistance. Since multistrand Litz wire can reduce the ac resistance, it is chosen to design the resonant coils. Litz wire (AWG44) with a number of 60 strands is used in transmitter coils, and with a number of seven strands it is used in receiver coils because of the size limit of the receiver coils. In the operating frequency (350 kHz∼850 kHz) of AWG44, the ac resistance is relatively low and a high Q factor can be achieved.

**Figure 2.**
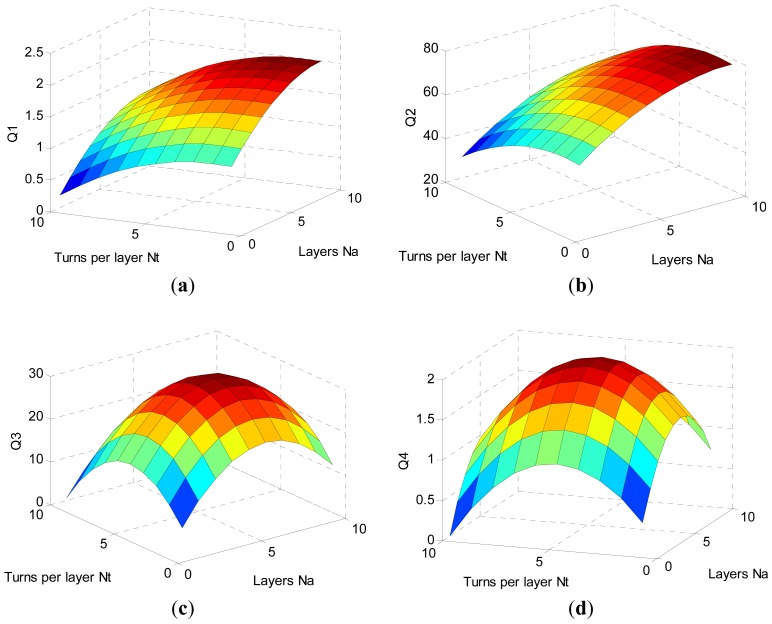
Optimized Q factor *versus* Na and Nt for every coil (**a**) Q_1_ *versus* N_a_, N_t_ for coil1; (**b**) Q_2_ *versus* N_a_, N_t_ for coil2; (**c**) Q_3_ *versus* N_a_, N_t_ for coil3; (**d**) Q_4_ *versus* N_a_, N_t_ for coil4.

Substituting Equations (1–3,5–7) into Equation (4), Matlab is used to calculate the tendency of Q *versus N_a_* and *N_t_*, as shown in Figure 2. The resonant frequency of the four coils is set at 650 kHz according to the operating frequency of AWG44 Litz wire, and the load resistance (*R_load_* = 50 Ω) for coil1 and coil4. According to the optimized Q and Equation (1), the self-inductance *L_a_* and a group of optimized geometry parameters are given in Table 1. The optimized inner diameters are set as coil1 of 34 mm, coil2 of 36 mm, coil3 of 14 mm, and coil4 of 16.5 mm.

### 2.2. Energy Transfer Efficiency Model

**Figure 3.**
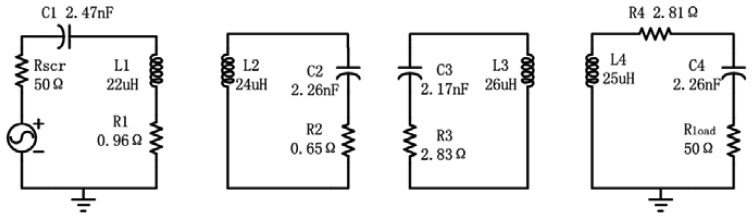
The equivalent circuit of power transfer system.

The CMT [8] and circuit theory [6] are commonly applied to analyze wireless power transfer systems. The CMT can be used to analyze the distribution of electromagnetic field properly at the far or middle range in the same resonator size and material, while it is not suitable for analysis of the four-coil system. Hence the resonant coupling model is built based on the resonant circuit theory. The equivalent circuit of power transfer system is proposed [6] and shown in Figure 3.

The KVL equation can be captured in the following matrix from:

(10) $$\left[\begin{array}{c} i_{1}\\ i_{2}\\ i_{3}\\ i_{4} \end{array}\right]={\left[\begin{array}{cccc} Z_{11} & Z_{12} & Z_{13} & Z_{14}\\ Z_{21} & Z_{22} & Z_{23} & Z_{24}\\ Z_{31} & Z_{32} & Z_{33} & Z_{34}\\ Z_{41} & Z_{42} & Z_{43} & Z_{44} \end{array}\right]}^{-1}\left[\begin{array}{c} U_{s}\\ 0\\ 0\\ 0 \end{array}\right]$$

(11) $$Z_{mn}=Z_{nm}=\{\begin{array}{c} R_{n}+j\omega L_{n}+\frac{1}{j\omega C_{n}}(m=n)\\ j\omega M_{mn}(m\neq n),M_{mn}=k_{mn}\sqrt{L_{m}L_{n}} \end{array}$$

where, Z_mn_, M_mn_, and k_mn_ are the circuit equivalent impedance, mutual inductance, and coupling factor between coilm and coiln. R_n_, L_n_, and C_n_ are the equivalent resistance, inductance and capacitance of the nth resonance circuit.

**Figure 4.**
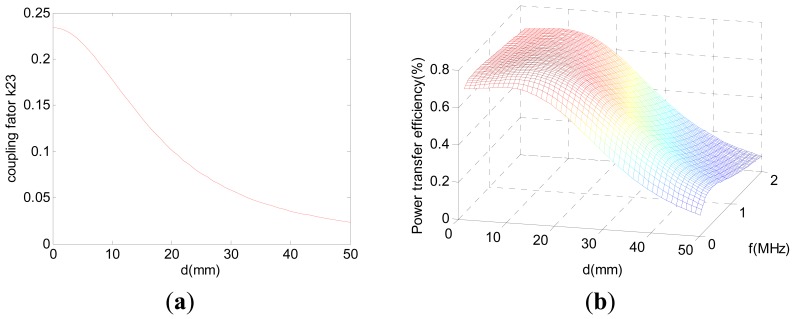
(**a**) Coupling factor k_23_ *versus* distance (k_12_ = k_34_ = 0.5, L_2_ = 25 μH, L_3_ = 28 μH); (**b**) Power efficiency *versus* distance and frequency.

The four coils are set to be resonant at the same frequency, *ω* = 1/√*L_n_C_n_, n* ∈ {1,2,3,4} and Z_mn_ = R_n_. For small driver and load coil inductance and relatively large distance between coil1 and coil4, coil1 and coil3, and coil2 and coil4, the cross coupling factor k_13_, k_14_, k_24_ can be neglected. The current in coil1 (i_1_) and coil4 (i_4_) can be calculated from Equation (10) and Q_n_ (Q_n_ = ωL_n_/R_n_). Therefore, the power transfer efficiency can be obtained as [6]:

(12) $$\eta =\frac{{i_{4}}^{2}R_{L}}{U_{s}i_{1}}=\frac{\left(k_{12}^{2}Q_{1}Q_{2})(k_{23}^{2}Q_{2}Q_{3})(k_{34}^{2}Q_{3}Q_{4}\right)}{\left[(1+k_{12}^{2}Q_{1}Q_{2})(1+k_{34}^{2}Q_{3}Q_{4})+k_{23}^{2}Q_{2}Q_{3}][1+k_{23}^{2}Q_{2}Q_{3}+k_{34}^{2}Q_{3}Q_{4}\right]}$$

from Equation (12), the coupling factor is the primary factor to improve power transfer efficiency. Based on equivalent circuit model, coupling factor between coil2 and coil3 can be calculated as:

(13) $$k_{23}=M_{23}/\surd L_{2}L_{3}$$

(14) $$M_{23}=N_{2}N_{3}M(a_{2},a_{3},d)$$

where N_2_ and N_3_ are the turns of coil2 and coil3. *M*(*a_2_,a_3_,d*) is the mutual inductance between two single coils (with radius of a_2_ and a_3_, distance d) according to Equation (2). According to the optimized coil geometry parameters according to the theoretical model in Table 1, the model of k_23_ can be calculated from Equations (13,14) and it is a function of distance. In additon k_12_, k_34_ can aslo be derived as 0.5 from Equations (13,14) under certain distance. Figure 4(a) plots the coupling factor k_23_ as a function of distance (d) between transmitter coils and receiver coils, and Figure 4(b) plots the power efficiency as a function of operating frequency (f) and distance (d) between transmitter coils and receiver coils. It is indicated that the maximum power transfer efficiency is achieved at 650 kHz. Therefore, based on the above optimization and analysis results, the best energy transfer efficiency is obtained at the working frequency of 650 kHz for this four-coil resonant power transfer system.

## 3. Peripheral Circuit Implementation

The peripheral circuit mainly includes the transmitter module and receiver module of the wireless power transfer system. The transmitter module is used to generate a power signal with a Class-E amplifier and the receiver module is used to process the receiver signal with a rectifier and voltage regulator circuit. The receiving voltage signal is converted to a stable output voltage of 3.3 V and a current of 10 mA at the distance of 2 cm.

### 3.1. Design of Class-E Power Amplifier

The Class-E power amplifier, known as the highest efficiency power amplifier, is used to reduce the power dissipation. In order to improve the efficiency of Class-E power amplifiers, three rules need to be considered when designing the Class-E: (1) Minimize the voltage across the device as the current flows through it; (2) Minimize the current flowing through the device when voltage exists; (3) Minimize the duration of any unavoidable condition in which appreciable current and voltage exist simultaneously [21].

**Figure 5.**
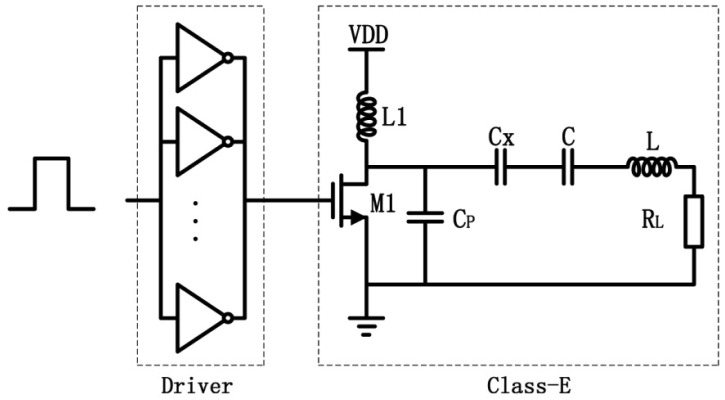
Schematic of the Class-E power amplifier and driving circuit.

The schematic of Class-E power amplifier introduced in this paper is shown in Figure 5. The basic Class-E power amplifier circuit is composed of MOS switch, RF choke, a parallel capacitor (C_p_), load network (LC) and load (R_L_) [22]. Parasitic capacitance of MOS drain increases C_p_ [23]. When analyzing the Class-E amplifier working principle, it is assumed that the on-resistance of MOS transistor is zero and the off-resistance of MOS transistor is infinite. Square wave is applied to the gate of MOSFET to control the MOS switch. When a high voltage is applied, the MOS switch is on and current flows through the MOSFET. The drain-to-source voltage is approximate zero. When a low voltage is applied, the MOS switch is off and the drain-to-source voltage equals to the voltage across C_p_. Because the magnetic resonant coupling between the transmitter coils and receiver coils will reduce the resonant frequency of the system, a tuning capacitance (Cx) in series with LC is added to adjust the resonant frequency point of the transmitting circuit.

At the resonant working state, the voltage across C_p_ may reach 40 V. Because the maximum drain-to-source voltage and the current of power MOSFET (IRF530) are 100 V and 17 A, the on-resistance of which is less than 110 mΩ, it is chosen as the MOS switch. Due to the large gate capacitance, a driver circuit is needed in front of the Class-E power amplifier. As driving stage, the inverter has the advantages of low cost and simple circuit structure. The power MOSFET can be driven effectively by twelve CMOS inverters (74HC04) [24]. The experimental result shows that the input signal of Class-E power amplifier is obviously improved and the rising time is 18 ns, so it can provide more power output to the wireless energy transfer system.

### 3.2. Design of Receiver Module

The receiver circuit module shapes the signal received from the receiver coils with the rectifier and voltage regulator circuit. It is used to provide a stable voltage source. This receiver circuit module is designed based on the CSMC 0.5 μm standard CMOS process with the Cadence simulation platform. It is composed of CMOS rectifier circuit and capacitor-less low dropout linear voltage regulator (LDO). In order to meet the demands of the area and power dissipation of the implantable chip, the rectifier chosen in this paper is compatible with the standard CMOS process and the LDO linear regulator has no off-chip capacitor. With the input power voltage changing from 3.5 V to 4.5 V, the LDO linear regulator can produce a stable 3.3 V power supply. When the output current of the LDO is 40 mA, it also has good stability and transient response.

**Figure 6.**
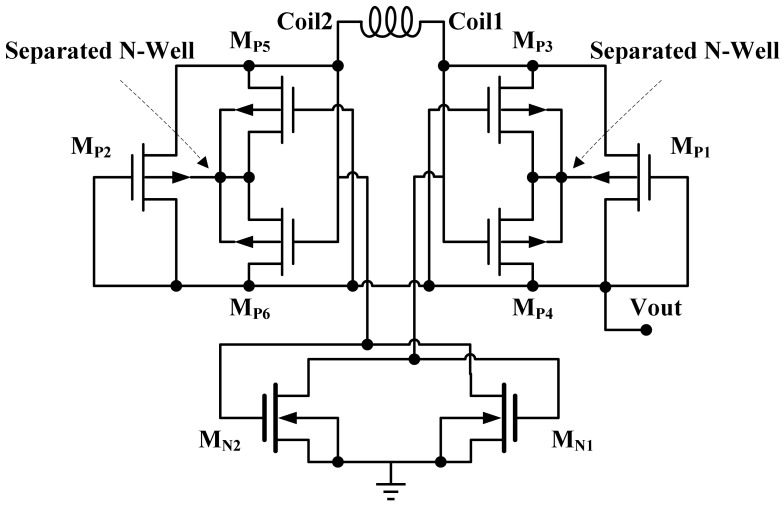
The diagram of the standard CMOS rectifier.

The structure of the rectifier [25] used in this paper is depicted in Figure 6. This rectifier does not dissipate too much power through substrate leakage current and rectifier dropout voltage, and does not increase the risk of latch-up. It is important to protect the circuit against latch-up and substrate leakage, because the source nodes of the rectifying pMOS transistors in Figure 6 are connected to the coil terminals, which have large voltage variations at high frequency. The separated N-wells are the nodes that increase the risk of substrate leakage current. In order to control each separated N-well voltage, the transistors M_P3_-M_P6_ are added to M_P1_ and M_P2_ to connect the N-well to V_out_, coil1, or coil2 whichever is at a higher potential. Besides, the higher substrate potential reduces the threshold voltage of M_P1_ and M_P2_. With the reduction of the threshold voltage, the power dissipation in the rectifier block decreases and the average rectified dc voltage available at the regulator input increases. Reducing the rectifier dropout voltage lowers the minimum receiver coil voltage, which in turn saves the required transmitted power or increases the maximum permissible coupling distance between the transmitter and receiver coils. The instantaneous voltage drop on the transistors of M_P1_ and M_P2_ can be found from:

(15) $$V_{GS}=\left|V_{DS}\right|=\left|V_{TH}\right|+\sqrt{\frac{2I_{D}}{\mu C_{ox}(\overset{W}{\;}/\underset{L}{\;})}}$$

where *I_D_* is the drain current, *μC_ox_* is the intrinsic transconductance, *V_TH_* is the transistor threshold voltage, and *W* and *L* are the transistor width and length. From Equation (15), the *W/L* value should be increased as much as the rectifier area consumption and its parasitic capacitance will permit us to lower the dropout voltage.

**Figure 7.**
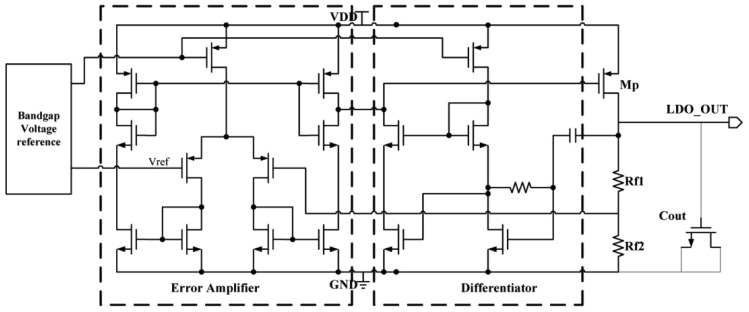
The structure of the capacitor-less LDO.

A capacitor-less LDO architecture [26] is presented in this section as depicted in Figure 7. It is composed of bandgap voltage reference, error amplifier, pass transistor, feedback network and a compensation circuit.

Since the capacitor-less LDO does not have the off-chip capacitor, a sound compensation scheme for both the transient response and alternating current stability is proposed. It is crucial to regulate the compensation scheme shown as the differentiator circuit in Figure 7. The relatively small on-chip output capacitor implemented by the MOS capacitance is about 100 pF. The output voltage of LDO voltage regulator is decided by the band-gap voltage reference and the feedback network. It can be found from:

(16) $$V_{\text{LDO\_OUT}}=\left(1+\frac{R_{f1}}{R_{f2}}\right)V_{\text{ref}}$$

where *R_f1_* and *R_f2_* are the resistors shown in Figure 7 and *V_ref_* is the bandgap reference voltage.

**Figure 8.**
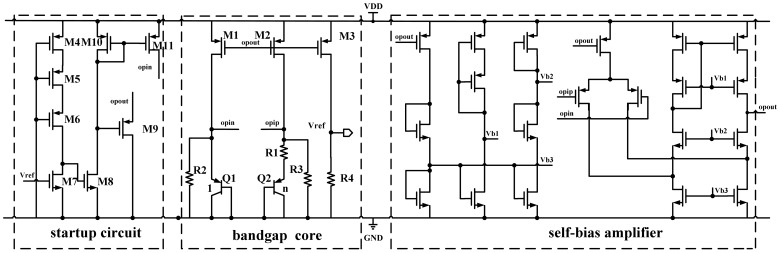
The schematic diagram of the bandgap voltage reference.

Bandgap voltage reference circuit uses the negative temperature coefficient of emitter-base voltage in conjunction with the positive temperature coefficient of emitter-base voltage differential of two transistors operating at different current densities to make a zero temperature coefficient reference. The structure [27] presented in this paper is depicted in Figure 8. The drain current of M1 and M2 is:

(17) $$I=\frac{V_{EB1}-V_{EB2}}{R_{1}}+\frac{V_{EB1}}{R_{3}}=\frac{V_{T}\ln(n)}{R_{1}}+\frac{V_{EB1}}{R_{3}}$$

where *V_T_* is the thermal voltage, *n* is the area ratio of Q2 and Q1, *R_1_* and *R_3_* are the resistors shown in Figure 8, and V_EB1_ and V_EB2_ are the emitter-base voltage of the transistor Q1 and Q2, respectively. If the size of M3 is equal to the size of M2 and the effect of channel-length modulation is neglected, we can obtain the output voltage of the bandgap reference:

(18) $$V_{\text{ref}}=IR_{4}=[\frac{V_{T}\ln(n)}{R_{1}}+\frac{V_{EB1}}{R_{3}}]R_{4}=\frac{R_{4}}{R_{3}}[V_{EB1}+\frac{R_{3}}{R_{1}}V_{T}\ln(n)]$$

where the temperature coefficient of V_EB1_ is negative and the temperature coefficient of V_T_ is positive. The compensation of the temperature coefficientsV_EB1_ and V_T_ is ensured by choosing values of n and of the R_3_/R_1_ ratio and the value of the output voltage is ensured by the *R_4_/R_3_* ratio.

Since this circuit has a dead (zero current) operating point, a startup circuit shown in Figure 8 is necessary to bring out the reference circuit from the dead operating point to its normal operating point. A self-bias amplifier is adopted in this circuit to reduce the circuit complexity and power consumption. This bandgap voltage reference provides a stable output voltage.

The receiver circuit module rectifies the alternating signal from the receiver coils. It is convenient to integrate the receiver circuit module by using the CMOS rectifier circuit and capacitor-less LDO voltage regulator. Besides, because of the integration of the receiver circuit module, the volume of the receiver circuit is reduced and the stability of the system is increased.

## 4. Measurement Results and Discussions

**Figure 9.**
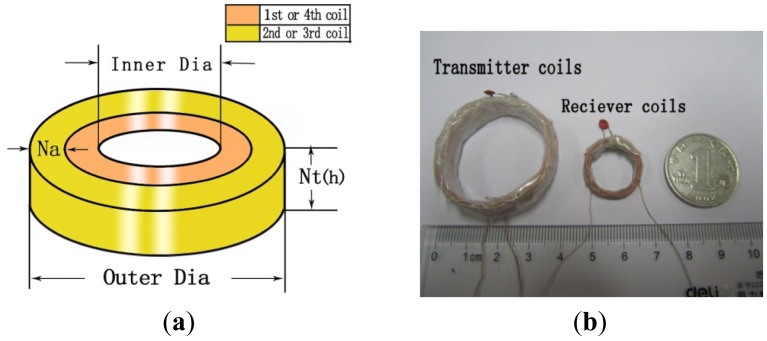
Description of the coils: (**a**) Description of the structure of the coils; (**b**) The size of the transmitter and receiver coils compared with a coin.

A detachable stent with variable dimensions was designed to accommodate different sizes of the coils, which can be removed to further reduce the volume of the coil. The structure of the coils is described in Figure 9(a), where coil2 is wrapped over coil1 and coil3 is wrapped over coil4. Sixty-strand and seven-strand Litz wire of AWG 44 are used to implement the transmitter coils (coil1, coil2) and receiver coils (coil3, coil4), respectively. Figure 9(b) shows the transmitter and receiver coils compared with a Chinese coin (diameter = 25 mm). Table 2 gives the geometric specifications and measured L and Q of the four coils according to the theoretically optimized results as shown in Table 1. The measured L and Q at the frequency of 742 kHz are also given in Table 2.

The implanted coils and the IC chip are integrated on a PCB board as the *in vivo* part and packaged as shown in Figure 1(b). Since Si_3_N_4_ is a good sealing material, a layer of 5000 Å Si_3_N_4_ is firstly deposited on the surface of the coils and PCB board by Plasma Enhanced Chemical Vapor Deposition (PECVD). Then the whole *in vivo* part is completely coated with approximate 15 um Parylene C. In order to improve the biological compatibility, PDMS is used for further and stronger packaging. The whole volume of the packaged system is 1 cm^3^. After packaging, the whole *in vivo* part is soaked in saline, and the sample is examined daily under a microscope. The soak lasted up for more than three months and the device was not eroded.

**Figure 10.**
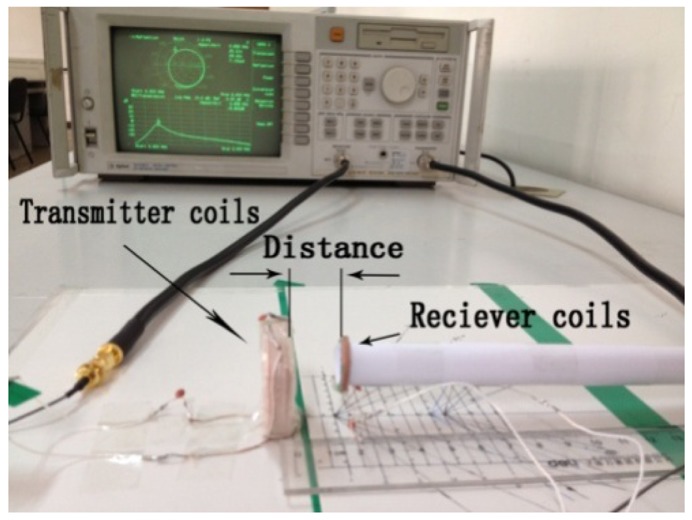
The experimental setup for the four-coil power transfer system.

As shown in Figure 10, a network analyzer (Agilent 8714ET) is used to measure the electrical specifications of the coils and the transfer efficiency of the wireless power transfer system. The coils' electrical specifications *i.e.*, L, Q, dc resistance, and ac resistance can be measured through the Smith Chart with the frequency sweeping from 300 kHz to 10 MHz. The power transfer efficiency can be obtained from the S-parameter:

(19) $$f(dB)=10\times Lg\left[S_{21}^{2}{/(1-\text{S}}_{11}^{2})\right]$$

**Figure 11.**
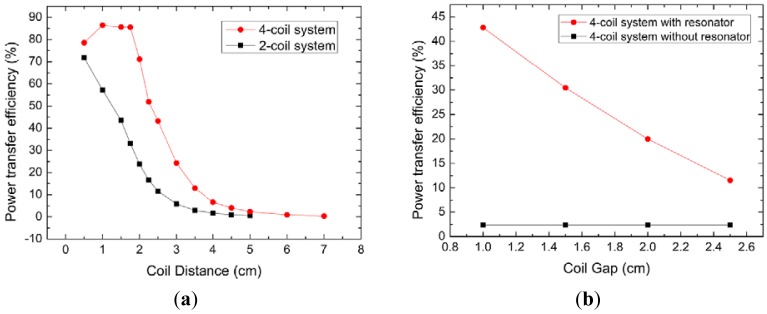
(**a**) η of two- and four-coil systems *versus* distance. (**b**) η of four-coil system with magnetic enhanced resonators under different gaps at the distance of 5 cm.

Figure 11(a) is the measurement results of the power transfer efficiency (η) *versus* different distance for two- and four-coil system. η of four-coil system is much higher than that of two-coil system in the measurement range (1 cm∼7 cm). It indicates that the four-coil power transfer system can meet different kinds of sensors' power demands. The power of 33 mW can be delivered at the distance of 2 cm. η of the four-coil system is 85% at the distance of 1.5 cm (r_m_/d = 0.9), which is much higher than 43% of the two-coil system. The highest η is 86% at the distance of 1 cm. What's more, η is little affected by the distance when the distance is less than 2 cm. Even if the distance is 3 cm, the efficiency is as high as 24%.

**Figure 12.**
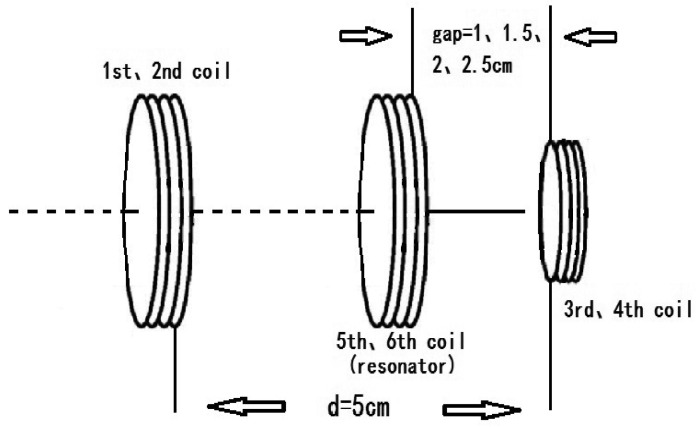
The diagram of adding magnetic enhanced resonators.

In order to improve the energy transfer efficiency at the same distance, two magnetic enhanced resonators (5th and 6th) are added as shown in Figure 12. The efficiency is improved with the increased number of enhanced resonators. Since the magnetic field of each coil is coupled in near-field, that is, the magnetic field is the fading field, the coupling strength becomes weaker as the transmission distance increases and the attenuation magnitude of the magnetic field is reduced by adding enhanced resonators. In addition, the coupling scope of the magnetic field is limited and when there are no extra enhanced resonators (5th and 6th), the coupling efficiency decreases with the increasing transmission distance. The coupling strength at the same distance is strengthened through adding enhanced resonators (5th and 6th) and the energy transfer efficiency (η) is improved. Figure 11(b) gives the experiment results of η for the system with and without the enhanced resonators at the same distance of 5 cm. Also, the position of the resonators has much influence on the efficiency. η *versus* the gap between enhanced resonators and receiver coils is measured. η of the four-coil system is below 2%, while it can be greatly improved by adding enhanced resonators. η of 43% can be obtained at the gap of 1 cm and it is increased by more than 20 times. The experiment results prove that the magnetic enhanced resonators can effectively improve the energy transfer efficiency.

**Figure 13.**
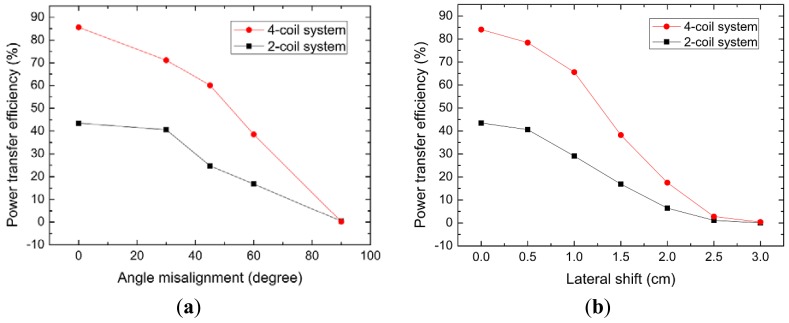
(**a**) η of two- and four-coil systems *versus* angle misalignments at d = 1.5 cm; (**b**) η of two- and four-coil systems under different lateral shifts at d = 1.5 cm.

In order to characterize the relationship between energy transfer efficiency and location parameters, η *versus* angle misalignment and lateral shift at the distance of 1.5 cm are measured and showed in Figure 13(a,b), respectively. The energy transfer efficiency of the two- and four-coil systems is both affected by location parameters obviously and it decreases with the angle misalignments and lateral shifts. Because of magnetic resonant coupling, η of four-coil system is higher than that of two-coil system in the whole range. Our experimental results also reveal that magnetic enhanced resonators can eliminate a part of influence brought by the location misalignment.

**Figure 14.**
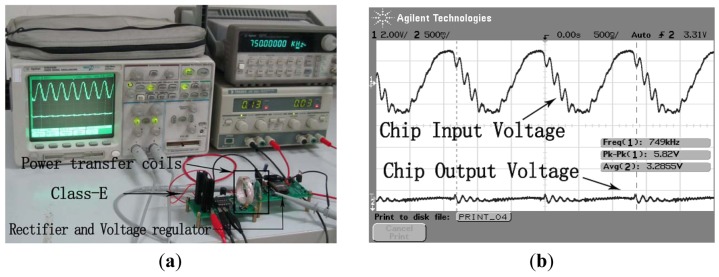
(**a**) The measurement platform of the whole system; (**b**) The measurement results of the chip input voltage and the chip output voltage.

The performance of the whole system is measured on the experimental platform as depicted in Figure 14(a). On the transmitter side, the Class-E amplifier is implemented based on Figure 5 and the input control signal is generated by an Agilent 33120A function generator. The resonant frequency of the Class-E amplifier is adjusted to 750 KHz, equal to the highest η frequency point of the four-coil power transfer system. On the receiver side, the chip, including the rectifier and LDO voltage regulator, follows the receiver coils. The output of the chip is loaded by a resistor of 330 Ω. When the distance between transmitter coils and receiver coils is 2 cm, a 3.28 V of output voltage is obtained. It is equivalent that a power of 33 mW is received. The wave of the chip input voltage and the chip output voltage is depicted as Figure 14(b). In addition, a power of more than 100 mW can be achieved when the distance is decreased, which can meet the power requirements of most reported biomedical implants consume such as artificial retina, intraocular pressure, and neural recording system.

## 5. Conclusions

A wireless energy transfer system with resonant four coils is presented in this paper. The whole system is composed of a Class-E amplifier, transmitter coils, receiver coils, and signal shaping chip which includes the rectifier module and LDO voltage regulator module. The electrical and geometrical parameters of the coils are theoretically optimized. The energy transfer efficiency is modeled and optimized based on the resonant circuit theory and it is measured based on the designed experimental setup. Experimental results show that the energy transfer efficiency of the resonant four coils is much higher than that of two coils. At the carrier frequency of 742 kHz, the measured coupling efficiency is 85% at the distance of 1.5 cm. The highest efficiency of 86% is obtained at the distance of 1 cm. Even if at the distance of 3 cm, the efficiency is as high as 24%. In addition, the power transfer efficiency can be improved by adding magnetic enhanced resonators. The system measurement results show that the receiving voltage signal is converted to stable output voltage of 3.3 V and a current of 10 mA at the distance of 2 cm. In addition, the output current is changed with the distance.

## Figures and Tables

**Table 1.** Optimized coil geometry parameters by theory model.

**Type**	**Coil Number**	**Outer Dia.** **(mm)**	**Inner Dia.** **(mm)**	**Turn/layers** **N_t_**	**Layers** **N_a_**	**L** **(μH)**	**Q (loaded)** **(650 kHz)**
Driver Coil	1	36	34	10	2	25	1.8
Primary Coil	2	38	36	10	2	26.5	75
Secondary Coil	3	16.5	14	6	5	26.4	29
Load Coil	4	19	16.5	6	5	24.8	2

**Table 2.** Specification of the four coils (Measured).

**Coil Number**	**Strand Dia.**	**Layers** **(Na)**	**Turns** **(Nt)**	**Inner Dia.** **(mm)**	**Outer Dia.** **(mm)**	**H** **(mm)**	**L** **(μH)**	**Q** **(at 742 kHz)**
1	0.05 mm	2	10	34	36	7	22	2
2	0.05 mm	2	10	36	38	7	24	173
3	0.05 mm	5	6	16	18	2	26	42
4	0.05 mm	5	6	14	16	2	25	2.17
